# Beyond words: investigating non-verbal indicators of collaborative engagement in a virtual synchronous CSCL environment

**DOI:** 10.3389/fpsyg.2024.1347073

**Published:** 2024-08-14

**Authors:** Loris T. Jeitziner, Lisa Paneth, Oliver Rack, Carmen Zahn

**Affiliations:** School of Applied Psychology, Institute for Cooperation Research and Development, University of Applied Sciences and Arts Northwestern Switzerland, Olten, Switzerland

**Keywords:** CSCL, non-verbal behavior, virtual synchronous learning, quality of collaborative group engagement, virtual learning groups, higher education

## Abstract

In the future of higher education, student learning will become more virtual and group-oriented, and this new reality of academic learning comes with challenges. Positive social interactions in virtual synchronous student learning groups are not self-evident but need extra support. To successfully support positive social interactions, the underlying group processes, such as collaborative group engagement, need to be understood in detail, and the important question arises: How can collaborative group engagement be assessed in virtual group learning settings? A promising methodological approach is the observation of students’ non-verbal behavior, for example, in videoconferences. In an exploratory field study, we observed the non-verbal behavior of psychology students in small virtual synchronous learning groups solving a complex problem via videoconferencing. The groups were videorecorded to analyze possible relations between their non-verbal behaviors and to rate the quality of collaborative group engagement (QCGE). A rating scheme consisting of four QCGE dimensions (Behavioral, Social, Cognitive, and Conceptual-to-consequential QCGE) was applied, and non-verbal behaviors during the task were coded based on related research literature. We quantitatively and qualitatively analyzed non-verbal behaviors as indicators of QCGE. The results show that groups use a wide range of non-verbal behaviors. Furthermore, certain non-verbal behaviors are significantly related to specific dimensions of QCGE. These results help to identify relevant indicators of QCGE in virtual synchronous learning settings and thus promote the development of advanced methods for assessing QCGE. Furthermore, the indicators can be discussed as possible anchors for supporting collaborative learning in virtual synchronous groups.

## Introduction

The future of learning in higher education will have to meet the new demands of future workplaces and changes in society as well as the new corresponding demands of students (Pelletier et al., 2023). Universities must evolve as organizations alongside current global trends and challenges in a complex world (Pelletier et al., 2023). In the time since the COVID-19 pandemic, trends in higher education include hybrid and remote learning settings as an emerging new normal or mainstream, with some universities now even offering purely remote programs (Pelletier et al., 2022). Furthermore, as collaboration is one of the key future skills in the workplace and therefore in higher education (Dishon and Gilead, 2021), group learning is becoming increasingly important. As a result of these current trends, students’ future learning will be more virtual (i.e., supported by digital tools) and group-oriented, and thus, computer-supported learning groups are becoming increasingly important in today’s higher education. Previous research on computer-supported collaborative learning (CSCL) has shown, on the one hand, that CSCL is a tool for improved learning of content and learner attitudes (Chen et al., 2018) and, on the other hand, a goal in its own right regarding the twenty-first-century skills of the future (Dishon and Gilead, 2021). This new reality of academic learning poses challenges. Although virtual group learning environments can sometimes be inspiring and stimulating, they may also be tedious. Solving study problems or writing or preparing for examinations together in groups can be socially challenging. Wilson et al. (2018) report, for instance, that students collaborating in groups may experience stress, interpersonal conflict, and unequal distributions of effort. Ferri et al. (2020) identified the challenge in virtual synchronous CSCL groups that the settings may reduce the social presence and the quality of social interactions between students. This is partly due to the reduced social cues in computer-mediated communication (e.g., Kiesler et al., 1984), resulting in uncertainties on the socio-emotional level. Adding to the problem is a certain neglect of social interaction quality at the higher education level in the sense that positive and productive social student interactions are often mistakenly taken for granted (Kreijns et al., 2003) and thus underrepresented in academic teaching concepts or lesson planning. Yet, positive social interactions in student learning groups are not self-evident as many failures in daily practice suggest. Or, to put it in other words, group dynamics need to be considered explicitly and supported actively in academic learning. This includes both socio-cognitive and socio-emotional process regulation (Järvelä and Hadwin, 2013), and success depends on complex regulatory processes, as was found in CSCL research (cf. Järvelä et al., 2019). In sum, there is a need and an opportunity to explore CSCL-relevant group processes in virtual synchronous environments. One important group process is the students’ collaborative engagement. This collaborative engagement is related to the group’s shared regulation (Lee et al., 2015) and based on their collaboration and social interaction (Sinha et al., 2015). More precisely, collaborative group engagement is a key element for CSCL, as it is central to the regulation process (Järvelä and Hadwin, 2013) and plays an important role in academic success (Liu et al., 2022). In consequence, to successfully support positive social interactions, the underlying group processes, such as group engagement, need to be understood in detail. From this, an important question arises: How can group engagement be assessed and supported in virtual synchronous group learning settings? One approach to assessing this multidimensional latent construct could be to explore different indicators on the behavioral level (e.g., non-verbal behavior and verbal communication). Within CSCL, the research concerning the multidimensionality of this construct has been limited (Sinha et al., 2015; Rogat et al., 2022; Xing et al., 2022), while to our knowledge approaches to using behavioral markers like non-verbal behavior are scarce.

Sinha et al. (2015) first introduced an approach to assess the construct of quality of collaborative group engagement (QCGE). The researchers defined collaborative group engagement as a multifaceted, shared, and dynamic core group process mediating group-level relationships between motivation, effort, and learning success (Sinha et al., 2015). They assessed QCGE using an observational approach, in which learning groups were rated at multiple time points during a collaborative learning task. Sinha et al. (2015) present a theoretical and methodological framework that distinguishes four dimensions: (1) Behavioral QCGE is conceptualized as the level of the group’s participation and effort invested in the ongoing task. Behavioral QCGE is necessary, but by itself, it does not ensure overall engagement, as students may complete activities without being engaged at the social and cognitive levels. Free riders (Salomon and Globerson, 1989) can reduce the quality of behavioral engagement by failing to cooperate and disengaging other group members. Thus, low Behavioral QCGE would be indicated by being disengaged from the task. (2) Social QCGE is conceptualized as the quality of the socio-emotional interactions, which can be observed by indicators of respectful and inclusive conversation as well as group cohesion and their degree of collaboration. High-quality Social QCGE, which frames tasks as joint efforts, enhances group cohesion, and facilitates task coordination, thereby increasing other dimensions of engagement (Sinha et al., 2015). High-quality Social QCGE also promotes shared understanding within the group (e.g., Dillenbourg et al., 2009). Low-quality Social QCGE, however, can lead to conflict and inequality (Salomon and Globerson, 1989). (3) Sinha et al. (2015) derived Cognitive QCGE from earlier frameworks on multidimensional school engagement (Fredricks et al., 2004). However, there is ambiguity in the naming convention for this dimension in previous research. The term “Cognitive engagement” in this framework could also be referred to as “Meta-cognitive engagement” as it consists of meta-cognitive processes such as task monitoring and socially shared regulation of cognition. More recent approaches to collaborative group engagement use dimensions such as “Meta-cognitive” rather than “Cognitive engagement (Rogat et al., 2022) or “Meta-cognitive engagement” as an additional dimension (Xing et al., 2022). In this study, we based our methodology on Sinha et al.’s (2015) QCGE framework, which is nuanced and theoretically sound. Therefore, we adopted the terms of the dimensions accordingly, which was useful for our purposes. Sinha et al. (2015) conceptualized Cognitive QCGE as the regulation of cognition and tasks in a collaborative group task. Reflecting the collaborative aspect of Cognitive QCGE, the active use of socially shared regulatory strategies (Järvelä and Hadwin, 2013) is central to this dimension. Thus, high Cognitive QCGE is evident in groups jointly regulating their task (i.e., planning and monitoring), whereas low Cognitive QCGE may show a focus on superficial aspects of the shared task. (4) Conceptual-to-consequential (CC) QCGE defines the groups’ indication of progressing toward the overarching task goal and how they achieve their learning goals by using evidence or sharing knowledge. As conceptualized by Sinha et al. (2015), CC QCGE is described as student groups that use subject-matter content to solve meaningful problems. High-quality CC is evidenced by groups that provide justifications for their solutions after critically considering alternatives and that connect their ideas to prior knowledge and the larger context of the problem. This can contribute to the development of conceptual understanding in computer-supported collaborative learning (CSCL) situations. The quality of these different dimensions determines the success of the group (Sinha et al., 2015). It is further emphasized that collaborative group engagement is dynamic within groups; hence, there are no “successful vs. failing learning groups” but rather high or low QCGE phases within groups’ collaborative processes that can (and must) be regulated (Isohätälä et al., 2020).

Although Sinha et al. (2015) originally applied their construct to a face-to-face setting, it was applied to a setting with virtual collaboration in higher education as well (Altebarmakian and Alterman, 2017). In their article, Altebarmakian and Alterman (2017) report that the virtual learning groups they observed over the course of a semester indicated a fluctuation of collaborative engagement during the stages of their group work, namely, that students showed higher content-based and individual engagement by the end of the semester. The authors argue that this rather individual engagement pattern may be related to the virtual nature of collaboration, which can be “difficult, unnatural, and awkward” (Altebarmakian and Alterman, 2017, p. 8). A possible explanation for this shift from collaborative to individual engagement may be reduced social presence. Social presence can be defined as the “feeling of togetherness” in computer-mediated communication (Hauber et al., 2005, p. 2). Even though modern computer-mediated communication offers high-quality audio-visual remote communication for virtual learning groups, the social presence of peers is still reduced (Hauber et al., 2005), and important social cues are missing concerning non-verbal behaviors (Mottet, 2000). For example, group members can only see their upper body or even only the face, fewer hand gestures, or whole-body movements.

In this context, research on non-verbal behavior has been considered important in both human–computer interaction (e.g., Turan et al., 2022) and CSCL research (e.g., Zahn et al., 2010; Rogat et al., 2022). This research often focuses on kinesics—the non-verbal language of the body, such as head movements, facial expressions, and posture (Burgoon and Dunbar, 2018)—as well as coverbal behavior, which is defined as gestures (e.g., hand gestures or eye contact) that follow speech (Kendon, 2000; Kong et al., 2017). To date, research has also focused on facial expressions (e.g., smiling or gaze direction) as a specific classification of non-verbal behavior that has served as the basis for many of the studies of non-verbal behavior (cf. Ekman, 2017). Systems have been developed that theoretically and empirically assign facial expressions to different affective states of people (e.g., FACS, Ekman and Rosenberg, 1997) as well as systems that link facial expressions to cognitive processes (FEC, Turan et al., 2022). In this paper, we refer to non-verbal behavior to include both coverbal behavior and facial expressions. Non-verbal behavior is important in collaborative group processes because it has several socio-cognitive and affective functions. It is part of a dynamic system of interactions within a social setting, and it underlies social processes such as social evaluations (Patterson, 2019). In addition, members of social groups, including learning groups, have a need to feel verbally and non-verbally validated by others (as conceptualized in social presence theory; Short et al., 1976). Furthermore, non-verbal actions such as head nodding, eye contact, and gestures can be used to communicate group engagement, participation, interest, and mutual reinforcement (e.g., Dunbar and Burgoon, 2005). Technological improvements have allowed researchers to successfully collect and analyze non-verbal behaviors, such as gestures and body posture, thereby increasing knowledge about group processes and collaborative behaviors (Schneider et al., 2021). For example, recent research has found that affirmative non-verbal behaviors associated with technology interactions, such as avatar nodding, have been shown to improve learning motivation and communication processes (Allmendinger et al., 2003), while eye-tracking technology has demonstrated the benefits of joint visual attention (i.e., mutual gaze at specific information on the screen in computer-mediated collaboration) on collaborative problem solving (Schneider and Pea, 2014). In particular, research has also shown that some non-verbal activities, such as hand gestures, smiling, eye contact, and nodding, are positively associated with learning outcomes as well as student and group engagement (e.g., Behoora and Tucker, 2015; Schneider et al., 2021; Paneth et al., 2024).

In other words, non-verbal behaviors provide important information about the emotions of the learners and the quality of their social relationships (e.g., Burgoon and Dunbar, 2018) and can therefore be valid indicators of important group processes such as the quality of collaborative group engagement (QCGE), as recent research suggests (Paneth et al., 2024). It is still open whether future virtual scenarios could be improved by automatic coding of such indicators during collaborative learning, e.g., for tailoring interventions to current processes. Therefore, the construct of QCGE, especially in virtual learning contexts where social cues are not so easy to discover (Ishii et al., 2019), and in relation to non-verbal indicators of it, needs more explanation and clarity from a theoretical viewpoint and new original empirical research. The goal of the study presented in this contribution is to provide new, original results to close this research gap. Precisely, the following two research questions are investigated: (1) How do student groups communicate non-verbally in a virtual synchronous learning setting? (2) Which non-verbal behaviors are indicative of which dimension of QCGE?

## Methods

### Sample and pilot study

The sample consisted of seven groups of three to four undergraduate students, resulting in *N* = 23 students. The participants, all psychology students, indicated a mean age of *M* = 27.36 and consisted of a majority of female participants (*N* = 19). Half of the participants indicated that they already knew all of the other group members. Twenty seven percent indicated that they knew one other group member, while 27% did not know anyone beforehand. Before conducting the main study, we carried out a pilot study to pre-test the instructions and the technical setup. Therefore, we invited two groups consisting of three members each. The pilot study was satisfying, and we proceeded with the main study.

### Context

The study took place in a realistic field setting during an online undergraduate course in applied psychology at a Swiss university. Adhering to common ethical standards (approved by the university ethics committee), students were free to participate in the study without any consequences related to their course performance (like course credits) or to any other factors when they decided not to. Participation in the study did not directly influence course success, and the teacher did not know who participated because the study was conducted independently by research staff (the teacher was not present at any time during the study). The students were recruited as part of the online course. In this online course, which was titled “Cooperation and New Media,” the students would learn about the hidden-profile task. Thus, students who signed up for the study could learn about the hidden-profile task by experiencing it themselves. For this study, we used a hidden-profile task that consisted of a “murder mystery.”

### Murder mystery task

The murder mystery task can be classified as a complex problem and hidden-profile paradigm. Problem-solving and the hidden-profile paradigm are popular methods in CSCL and group research. In such a task, groups are presented with general information (shared information), while each member of the group is provided with additional information (unshared information), that the other group members are not provided with. Thus, the groups need to share their knowledge to optimize their decision-making (Sohrab et al., 2015). The learning objectives of the task consisted of two aspects: First, students could learn about success factors and barriers to virtual cooperation, which contribute to their professional competencies in work and organizational psychology. Precisely because they had to gather individual and group-related experiences in exchanging information and making decisions in virtual groups, then they could reflect on their experiences and integrate this new experiential knowledge with aspects of psychological theory. Second, they learned about the core statements of the theoretical model behind the hidden profile through a practical application, which makes the theoretical and practical implications easier to understand.

### Procedure

Participants who signed up for the main study were first informed about the study, its contents, and its objectives by the lecturer. They were informed that they would have the choice to participate in a study on virtual cooperation in the upcoming course. As the task was related to the course content (“learning by doing”), participation in the study was a voluntary learning opportunity. Students who chose to participate in the study (i.e., *N* = 23 out of a total of 85 students) had 2 h of the following regular course available to them; students who chose not to participate could use these 2 h for themselves or for studying. Study participants then received further instruction from academic staff. They first received an informed consent form with all relevant information about the study, the voluntary nature of participation in accordance with current ethical guidelines (see above approved ethics vote), and the fact that participation was independent of performance assessment in the module. Because the study was conducted online using a videoconferencing tool, students provided consent via a secure online form. After completing the consent form, they were given detailed instructions on how to proceed with the study. To this end, students were first given extensive instructions on how to proceed in the study and were given the opportunity to ask questions. In addition, group members were assigned different roles during the task: breakout room manager (i.e., securing the gallery view within the breakout session and video recording the online collaboration), whiteboard manager (i.e., ensure the functioning and order within the digital whiteboard), and discussion leader (i.e., keeping track of time and leading the discussion if it gets stuck). As part of the individual preparation phase within the breakout sessions, the students were given detailed instructions on how to carry out their specific roles. The purpose of these roles was to meet the technological requirements of collaborating with the digital whiteboard and video conferencing tool, to ensure time management, and to avoid diffusion of responsibility (Tosuntaş, 2020). We did not explicitly include collaborative roles for the purpose of scripting and scaffolding (Fischer et al., 2013; Vogel et al., 2017), as this was not the main scope of our study. Students were then divided into random breakout groups and asked to solve the “murder mystery” task. Within the breakout rooms, groups were provided with shared and non-shared information about a fictitious murder case and were given 20 min to individually set up their workstations and read the instructions as well as the (partially non-shared) information about the “murder mystery.” Then, the group task and the actual information exchange in the virtual group started, and they were given 30 min to reach a consensus and find the “murderer.” This part of the study was video recorded, as it was the focus of our observation. As they worked on a solution, they used a digital whiteboard to take notes and document their progress. Finally, the groups were instructed to decide who the murderer was, based on the evidence they had gathered. After completing the task, student groups were instructed to finish and save the video recording independently. They were then given a short online questionnaire to complete individually, asking for demographic information, acquaintance with group members, previous experience with virtual collaboration, and other variables such as enjoyment and interest (IMI, Ryan, 1982), subjective satisfaction with group outcome and learning success, and self-rated collaborative group engagement (QCGE, Paneth et al., 2023). Subsequently, the groups reconvened in plenary, and the study guides solved the “murder mystery.” As part of the regular post-study course, students were then debriefed and given the opportunity to reflect on their learning gains from the hidden-profile tasks.

### Instruments and materials

The units of interest in this study were the 1-min sequences of collaboration that resulted in *N* = 197 sequences that were observed, rated (QCGE), and coded (non-verbal behaviors). We used a *coding* scheme for coding the non-verbal behaviors of the study participants in the learning groups and a *rating* scheme to rate the QCGE in the groups. The schemes will be described in the following sections.

### Non-verbal behavior coding scheme

To collect non-verbal behavior data like students’ gestures and facial expressions during video conferences, a non-verbal behavior coding scheme was developed and applied. We developed the coding scheme based on an iterative method consisting of a deductive and an inductive approach. In the initial deductive approach, we inferred non-verbal indicators of engagement based on literature about non-verbal communication and social interaction (e.g., Husebø et al., 2011; Mahmoud and Robinson, 2011; Schneider and Pea, 2014; Behoora and Tucker, 2015; Burgoon and Dunbar, 2018; Rack et al., 2018; Noroozi et al., 2021). For instance, Behoora and Tucker (2015) found that propping the head can signal boredom and therefore presumably disengagement. Subsequently, we scanned the video data for additional frequently occurring non-verbal behaviors of participants completing the study to inductively enhance the coding scheme. The coding scheme was then tested by two trained raters, applying it to a video recording of one learning group from our study. To yield interrater reliability, we calculated *intraclass correlation coefficients* (ICC) for each non-verbal behavior in the coding scheme following the instructions from Koo and Li (2016). The ICC estimates and 95% confidence intervals were calculated by applying the R package irr (Gamer et al., 2019) based on a single-rating (*k* = 2), absolute-agreement, two-way fixed-effects model ICC (2, 1). The ICC was calculated for each code in the coding scheme, and the results are presented in Table 1. The two raters indicated moderate-to-excellent ICC for all but one code. We excluded that code (i.e., scratching of the head or neck area), as that specific ICC indicated poor interrater reliability (ICC = 0.433). The final coding scheme consisted of seven codes (see Table 1). As the QCGE rating scheme was divided into 1-min sequences (see below), we aligned the coding of non-verbal behaviors by also creating 1-min sequences and then asking the trained coders to provide their behavior codes following the instructions. The frequencies of the codes were then counted for each person in each group. This resulted in a dataset with N = 636 rows, consisting of aggregated frequencies of non-verbal behavior for each person within each 1-min sequence, as well as the group ratings of QCGE for each dimension within each 1-min sequence.

**Table 1:** Coding scheme of non-verbal behavior, mean frequencies and standard deviation per sequence, intraclass correlation coefficients, and confidence intervals.

				95% CI	
Code	Description	*M* (SD)	ICC	Lower	Upper
Propped-up head	Face or chin must be propped on hand	2.02 (1.34)	0.937	0.903	0.959
Hand in front of face	Hand in front of face but no propping	1.70 (1.36)	0.856	0.784	0.905
Head nodding	Vertical nod, no horizontal shake	4.54 (3.28)	0.661	0.515	0.769
Leaning forward	Moving the upper body toward the screen	1.84 (1.26)	0.550	0.375	0.686
Gesturing	All movements with hands, including pointing	0.82 (1.02)	0.855	0.783	0.905
Laughing	Smiling and laughing with and without sound	2.00 (2.34)	0.902	0.852	0.936
Changing of the seating position	Moving the seating position in any direction	2.23 (1.59)	0.619	0.445	0.744

### Quality of collaborative group engagement rating scheme

To measure QCGE, an observation-based rating scheme was used, originally developed by Sinha et al. (2015), based on their definitions of the four dimensions of collaborative group engagement (Behavioral, Social, Cognitive, and CC). As demonstrated by Sinha et al. (2015) in their study, this rating scheme distinguishes between three levels of QCGE (low, moderate, and high) on each dimension. The rating procedure consists of segmenting transcribed student group conversations into short sequences (time-based) and then asking trained raters to rate QCGE in each of these sequences as low, moderate, or high for each QCGE dimension (see Table 2). In our study, the transcript was segmented into 1-min sequences, resulting in a total of *N* = 193 sequences over all groups. Our rating scheme thus adheres to the original QCGE definitions by Sinha et al. (2015), on the one hand, and, on the other hand, was adapted to the task the students were working on. The rating instructions are presented in Table 2. The rating scheme was tested by two raters applying it to the transcripts of one group. For interrater reliability, we followed the same approach as described above concerning the non-verbal coding scheme. The ICC was calculated for all four QCGE dimensions and is presented in Table 3. The two raters indicated good to excellent ICC for all four dimensions.

**Table 2:** Definition and rating instruction by QCGE dimension, adapted from Sinha et al. (2015).

Dimension	Rating instruction
Behavioral	**High**: No off-task behavior **Moderate**: One member is off-task **Low**: More than one member is off-task
Social	**High**: Equal contribution, respectful tone **Moderate**: One or two members dominate the discourse, respectful tone **Low**: One member dominates discourse, disrespectful tone
Cognitive	**High**: Group indicates a thorough plan which represents the solution to the task (i.e., how to find the murderer) and task monitoring (i.e., timekeeping) **Moderate**: Group indicates an incomplete plan or only task monitoring (i.e., timekeeping) **Low**: Group indicates no structure in their task approach and no task monitoring
Conceptual-to-consequential (CC)	**High**: Evidence is used in discourse; knowledge is shared; discourse is on finding the murderer **Moderate**: Discourse is about connecting knowledge; use of evidence is inconsistent **Low**: Discourse is based on declarative knowledge (facts, no interpretation and sharing of knowledge); use of evidence is inconsistent

**Table 3:** Quality of collaborative group engagement (QCGE) rating scheme, mean rating and standard deviation per sequence, intraclass correlation coefficients, and confidence intervals.

				95% CI	
Code	Description	*M* (SD)	ICC	Lower	Upper
Behavioral	On-task/off-task behavior	2.91 (0.29)	1	NA	NA
Social	Inclusion, respectful interaction, collaboration	2.84 (0.37)	0.792	0.591	0.900
Cognitive	Planning, structuring, task monitoring	1.37 (0.52)	0.853	0.701	0.931
CC	Use of evidence, connection of shared knowledge, working on task goal	2.00 (0.79)	0.751	0.520	0.880

For Behavioral QCGE, the agreement between the two raters was perfect; therefore, the confidence intervals and *F*-test results indicate not applicable (NA).

### Quantitative and qualitative data analysis

**Figure 1:**
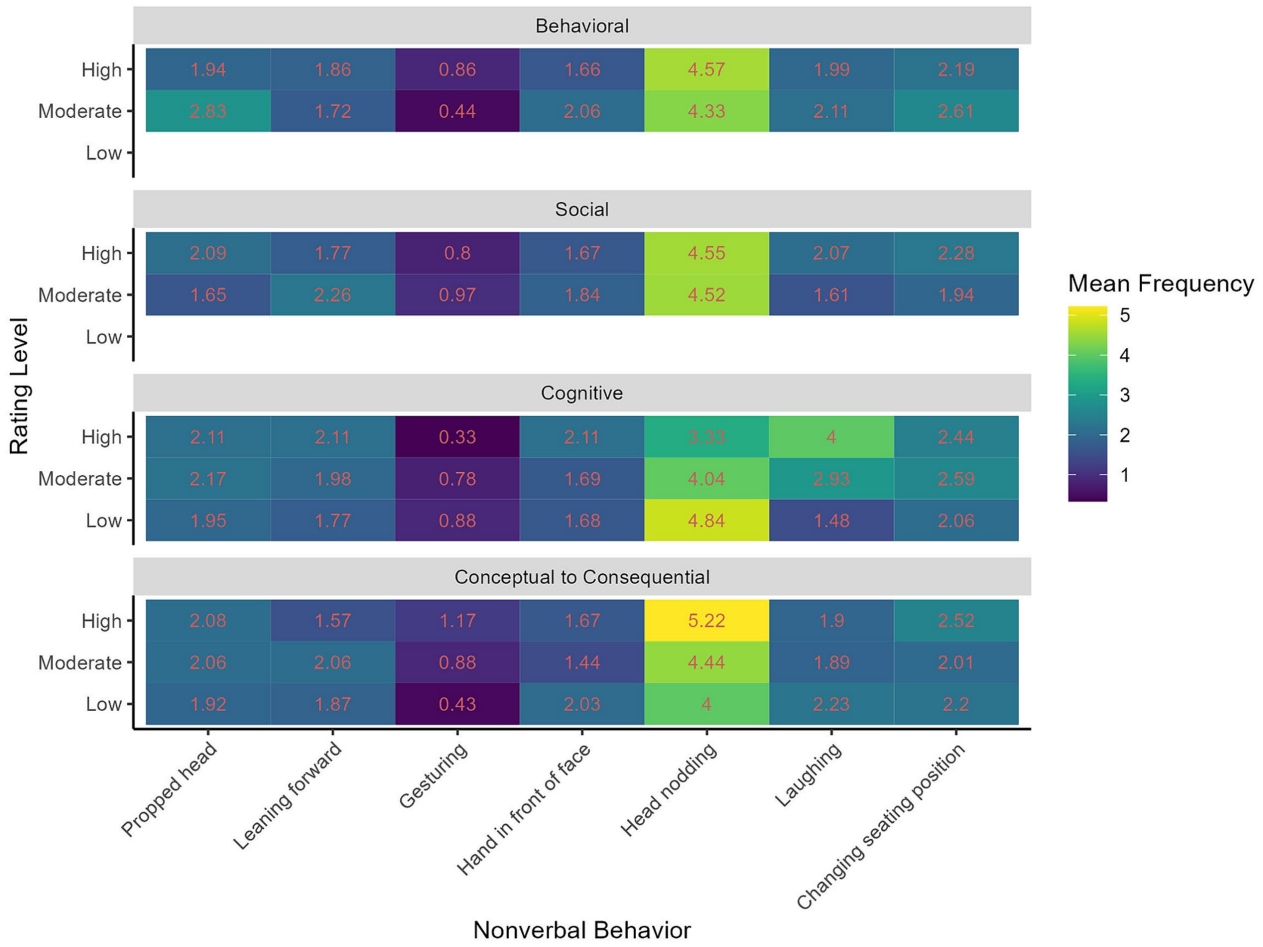
Mean frequencies per 1-min sequence for each QCGE dimension level and non-verbal behavior.

**Figure 2:**
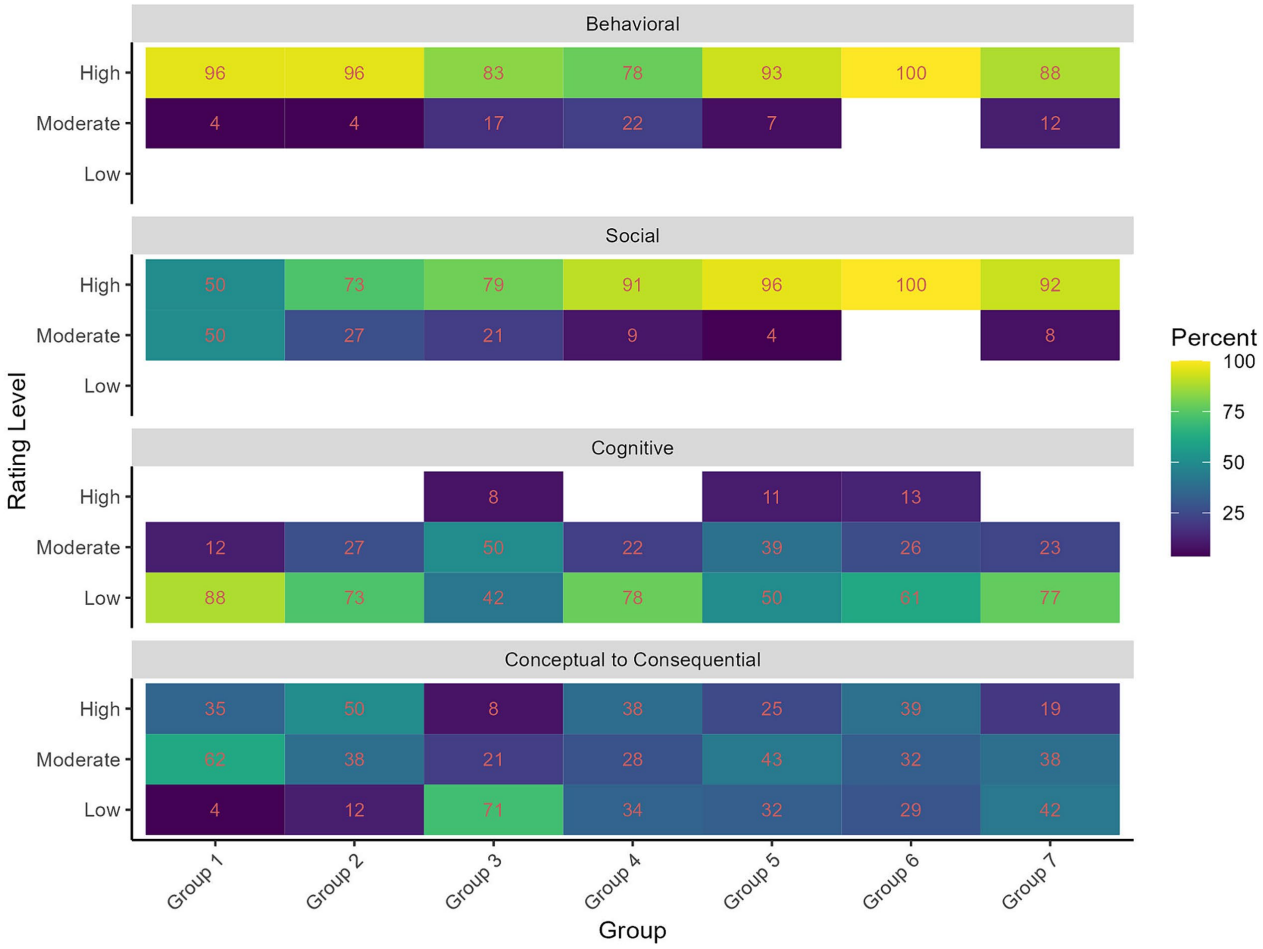
QCGE rating distribution categorized by group and QCGE dimension.

In order to answer the research questions, we analyzed our data according to the following mixed-method approach: To get a general insight into our data and answer the first research question, we visualized the variation of the data for the frequencies of non-verbal behavior (see Figure 1). To answer the second research question, we visualized the QCGE ratings and compared the frequencies of non-verbal behavior with the fluctuations of QCGE by visualization (see Figure 2) and statistical modeling. Since the data was based on repeated measures of frequencies of observed non-verbal behavior and hierarchical (i.e., participants were nested in groups), we calculated mixed-effects models with the ratings of each QCGE dimension for each minute as the dependent variable. As the QCGE rating scheme produced an ordinal structure, we ran cumulative-link mixed models with the R package ordinal (Christensen, 2019). We fitted the data with four models, one for each dimension of QCGE. For each model, we defined all the non-verbal behavior frequencies as fixed effects. Since we repeatedly assessed the frequencies over each 1-min sequence and aimed at controlling for sequence effects, we added the sequence as a random intercept. We also modeled the participants and groups with random intercepts and nested the participants in each group to control for individual- and group-level random effects. To counter convergence problems, the approach recommended by Bates et al. (2015) was followed by calculating the most complex model first and iteratively simplifying and comparing the models. For each dimension, we initially calculated the full model, namely with each frequency of non-verbal behavior per sequence as a fixed effect. From there on, to simplify the model, we removed insignificant (*α*-level = 0.05) non-verbal behavior fixed effects. We then compared the initial model with the simplified one and continued with the better fit. From there on, we tested the random effects for each model and removed random effects with no variance.

To highlight and further explore the relationships between QCGE and non-verbal behaviors, we conducted a qualitative analysis based on video analysis methods (Zahn et al., 2021) and case analysis methods (Koschmann and Schwarz, 2021). For this purpose, we scanned qualitative data (i.e., transcripts, QCGE ratings, and non-verbal behavior codes) for illustrative sequences (i.e., cases) in which the relationships found between non-verbal behaviors and QCGE dimensions were significant for the purpose of case illustration. More specifically, we selected 10 sequences with the most frequent correlated non-verbal behaviors and 10 sequences with the least frequent correlated non-verbal behaviors. For example, for the positive correlation between high CC QCGE and head nodding (see Results section), we selected 10 sequences in which CC QCGE was of high quality and in which head nodding occurred most frequently. Conversely, for this dimension, we selected 10 sequences in which CC QCGE was low quality and head nodding occurred least frequently. We then carefully reviewed these selected sequences within the recorded video material and made notes on them. This allowed us to fully describe and analyze the narrative within the sequence in terms of the relationships between the QCGE dimensions and the corresponding non-verbal behaviors. Finally, we prepared them as case illustrations, highlighting and describing the interplay between the non-verbal behaviors and the quality of the QCGE dimensions to enhance the results found by our quantitative analysis. Exemplary sequences were then selected for each QCGE dimension to be illustrated in the results section.

## Results

The following analyses were conducted with version 4.2.1 of R (R Core Team, 2023) in the R Studio environment (RStudio Team, 2023).

### Non-verbal communication

We counted the total frequencies across all non-verbal behaviors (cf. Table 1) and plotted the mean frequencies per non-verbal behavior and QCGE as well as the QCGE rating level (1 = low, 2 = moderate, 3 = high) in Figure 1. We also present the mean frequencies and standard deviation per 1-min sequence in Table 1. Figure 1 shows the differences in mean frequencies between the non-verbal behaviors, the four QCGE dimensions, and the rated QCGE levels. Results show, on a descriptive level, that head nodding occurs more frequently than gesturing. All other non-verbal behaviors occurred in a similar quantity per sequence.

### Non-verbal behavior as indicators of QCGE

Figure 2 illustrates the distribution of the QCGE ratings for each group and QCGE dimension. The results suggest that the groups received similar ratings of QCGE for the dimensions of Behavioral and Social QCGE. Across groups, the mean ratings (low = 1, moderate = 2, and high = 3) for Behavioral and Social QCGE are higher than Cognitive and CC QCGE (see Table 3). Furthermore, the standard deviations over all groups for Behavioral and Social QCGE are smaller than Cognitive and CC QCGE (see Table 3). The lower standard deviations in the ratings are apparent in Figure 2 for Behavioral and Social QCGE, where it is indicated that for both dimensions there were no low ratings, and in general, low variance with a skew to high ratings.

The results of the descriptive statistics and statistical modeling answered our question about whether the coded non-verbal behavior is indicative of QCGE. In Figure 1, the changes in mean frequencies per rating level show the direction of the relationship between non-verbal behavior and the QCGE rating. For instance, the mean frequency of head nodding increases with higher ratings for CC QCGE. On the other hand, the mean frequencies of the hand in front of the face do not follow a linear relationship with CC QCGE ratings, as the frequency for the sequences with a moderate rating (*M =* 1.44) was lower than for the high (*M =* 1.67) and low (*M =* 2.03) ratings. Further findings are described below in the sections regarding indicators of QCGE.

The results of the cumulative-link mixed model for each of the four dimensions suggest that both random effects of the group and sequence level turned out to enhance the fit of the model. For all four QCGE dimensions, the random intercept term of the individual participant that was nested in the group indicated no variance, and we therefore removed it.

### Indicators of behavioral QCGE

The results of the cumulative-link mixed model for Behavioral QCGE (cf. Table 4) suggest that groups where participants were more likely to prop their heads indicated lower Behavioral QCGE than groups with less head propping. The odds ratios indicate a 36% increase in the odds of being rated lower on Behavioral QCGE for each one-unit increase in head propping. Moreover, the random effects seem to explain parts of the variability of the model, supporting the inclusion of random intercepts of the group and the sequence.

**Table 4:** Cumulative-link mixed model for behavioral QCGE.

Predictors	Odds ratios	CI	*p*
Propped-up head	0.64	0.45–0.91	0.013
**Random effects**			
σ^2^	3.29		
τ_00 Sequence_	2.91		
τ_00 Group_	1.51		
ICC	0.57		

CI, confidence interval; σ^2^, overall random effect estimated variability; τ_00_, random intercept variability for group and sequence; ICC, intraclass correlation coefficient.

A qualitative exemplification of the negative relationship between Behavioral QCGE and a propped-up head is presented in Table 5. In the sequence at minute 17 of Group 4, members indicate higher frequencies of propped-up heads and only a moderate rating of Behavioral QCGE. This sequence was rated moderate on Behavioral QCGE quality, as evidenced by the fact that only two of three group members are on-task and that longer pauses occur during which group members do not appear to be on-task. At the beginning of this sequence, only Group Member 1 participates actively in the conversation and thus seems to be on-task, sharing information about the murder case. Only after a break and in the second half of the sequence, Group Member 2 joins the conversation by adding information about the murder case. All three group members propped up their heads, appearing either bored or overwhelmed by the task, which seems to highlight the moderate-quality Behavioral QCGE.

**Table 5:** Section of action transcript of Group 4.

Time	Group Member 1	Group Member 2	Group Member 3
17:13.8	[propped-up head] and then… so that would be a motive somehow, and also that they always have arguments… and with Mr… Hölscher, the money would simply be the motive, or rather he probably did not want to kill him, maybe he just wanted to steal his wallet, but… it then degenerated to an extent….		
17:23.1	[propped-up head]		
	PAUSE	PAUSE	PAUSE
17:26.5			[propped-up head]
17.38.1		I still have the statement of Marion Schmidt	
17:38.4		[propped-up head]	
17:40.3	mhm…		
17:41.2		She said she heard noise at 6.40 a.m.	

### Indicators of social QCGE

The results of the cumulative-link mixed model (cf. Table 6) suggest that no non-verbal behavior relates significantly to Social QCGE. However, from all non-verbal behavior, head nodding indicates a similarly high odds ratio compared to other models we present. Moreover, the qualitative analysis indicates face validity of the relationship between head nodding and Social QCGE. Thus, we included this model in the results. The odds ratios indicate a 20% increase in the odds of being rated higher on Social QCGE for each one-unit increase in head nodding. Moreover, the random effects seem to explain parts of the variability of the model, supporting the inclusion of random intercepts of the group and the sequence.

**Table 6:** Cumulative-link mixed model for Social QCGE.

Predictors	Odds ratios	CI	*p*
Head nodding	1.20	0.99–1.46	0.070
**Random effects**			
σ^2^	3.29		
τ_00 Sequence_	1.07		
τ_00 Group_	3.31		
ICC	0.57		

CI, Confidence Interval; σ^2^, overall random effect estimated variability; τ_00_, random intercept variability for group and sequence; ICC, intraclass correlation coefficient.

For example, during a sequence of Group 2 (cf. Table 7), Social QCGE was high, and the group indicated high frequencies of head nodding. As can be seen in the conversation among group members, the group gathers information about the murder case, each reinforcing the other verbally (i.e., “mhm,” “yes,” “correct”) as well as non-verbally (i.e., head nodding). What is further noticeable in this sequence is that group members tend to complete the sentences started by other group members (see the sequence from min. 02:19.0). This shows responsiveness and thus a high-quality Social QCGE (Sinha et al., 2015). This responsiveness is subsequently supported by frequent nodding. Moreover, a nod from one group member is often followed by nods from the other group members (see min. 02:27.2; 02:43.0; 02:49.6). This also seems to have a reinforcing effect, and the nod is often automatically perceived as a “yes” and thus as confirmation of one’s own statement, whereupon conversation continues.

**Table 7:** Section of action transcript of Group 2.

Time	Group Member 1	Group Member 2	Group Member 3
02:19.0	mhm… And then it’s also about the tire tracks….		
02:27.2		[head nodding]	
02:27.3	[head nodding]		
02:28.1		mhm…	
			… that were found.
02:29.9		mhm…	
02:30.0			… which were not yet there on Friday because it was raining….
02:33.4	correct		
02:36.3		right	
02:39.4	and he left after informing Mrs. Schmidt…		
02:43.0			[head nodding]
02:44.4		[head nodding]	
02:47.2			mhm…
02:47.3	… about and um he left then, where the emergency doctor came		
02:49.6			[head nodding]
02:50.1		[head nodding]	
02:54.0	[head nodding]		

### Indicators of cognitive QCGE

The results of the cumulative-link mixed model for Cognitive QCGE (cf. Table 8) suggest that groups where participants were more likely to laugh or lean forward received significantly higher ratings on the Cognitive QCGE dimension, than groups with less laughing or leaning forward (cf. Figure 1). The odds ratios indicate a 64% increase in the odds of being rated higher on Cognitive QCGE for each one-unit increase in laughing and a 42% increase in the odds of being rated higher on Cognitive QCGE for each one-unit increase in leaning forward. Concerning head nodding, the results suggest that there is a negative relationship to Cognitive QCGE (cf. Figure 1). The odds ratios indicate a 16% increase in the odds of being rated lower on Cognitive QCGE for each one-unit increase in head nodding. Moreover, the random effects seem to explain parts of the variability of the model, supporting the inclusion of random intercepts of the group and the sequence.

**Table 8:** Cumulative-link mixed model for cognitive QCGE.

Predictors	Odds ratios	CI	*p*
Head nodding	0.84	0.72–0.99	0.033
Leaning forward	1.42	1.08–1.88	0.013
Laughing	1.64	1.31–2.05	<0.001
**Random effects**			
σ^2^	3.29		
τ_00 Sequence_	1.59		
τ_00 Group_	1.09		
ICC	0.45		

CI, confidence interval; σ^2^, overall random effect estimated variability; τ_00_, random intercept variability for group and sequence; ICC, intraclass correlation coefficient.

The relationship between laughing and Cognitive QCGE is exemplified in Table 9. In this sequence of Group 6, we found high frequencies of laughing as well as a high Cognitive QCGE. It is evident here that the four group members are consistently engaged in task monitoring and planning. When Group Member 1 checks whether the group have gathered all relevant information at the beginning, Group Member 4 reacts by reading her information again, Group Member 3 reminds the group about the remaining time, and Group Member 2 proposes a plan of action, all of which can be characterized as task monitoring activities. In addition, they seem to get along well with each other, as evidenced by multiple laughing from all group members. Here, laughing seems to have a trust-building and loosening function in the sense of an icebreaker. This trust-building seems to motivate all group members to make suggestions for the further planning of the solution of the task and also to critically question the previous task monitoring and to adapt it (see sequence from minute 23:21.2). Group Member 2 expresses that she finds it difficult to collect information because of its arrangement and Group Member 4 responds with a self-critical and reflective statement that she could have done a better job of writing down the information on the shared whiteboard.

**Table 9:** Section of action transcript of Group 6.

Time	Group Member 1	Group Member 2	Group Member 3	Group Member 4
23:10.5				[laughing]
23:14.1		[laughing]		
23:14.8			[laughing]	
23:15.2			We have exactly 3 min left…	
23:15.2			[laughing]	
23:17.8			[Confused chatter]	
23:18.3		Come on now just say something		
23:18.3		[laughing]		
23:14.8	[laughing]			
23:19.1				I did not write down that much either…
23:21.2				[laughing]
23:25.8		It’s just difficult now, because everything is so scattered, you know, it would be more practical if you could put it down and…		
23:32.6	mhm…			
23:34.2		but this way it’s slide by slide…		
23:38.3				umm I could have written it down a little bit better, yeah….
23:40.6				[laughing]

Furthermore, the relaxed atmosphere in this sequence of Group 6 allows for jokes about working together, which in turn builds trust. The relationship between laughing and Cognitive QCGE could be explained here by the fact that task monitoring is usually a rather serious matter, and, depending on the group constellation, group members do not always courageously integrate this element into group work. One does not always make oneself popular if one strictly monitors and corrects the processing of tasks. However, if there is a lot of laughing and thus a development of trust and compassion, this can encourage the group members to also include somewhat more serious and perhaps more unpleasant elements, such as task monitoring.

### Indicators of CC QCGE

The results of the cumulative-link mixed model for CC QCGE (cf. Table 10) suggest that groups where participants were more likely to nod their heads received significantly higher ratings on the CC Engagement dimension than groups with less head nodding. For head nodding, Figure 1 corroborates this linear relationship. The odds ratios indicate a 14% increase in the odds of being rated higher on CC QCGE for each one-unit increase in head nodding.

**Table 10:** Cumulative-link mixed model for CC QCGE.

Predictors	Odds ratios	CI	*p*
Head nodding	1.14	1.00–1.29	0.048
Leaning forward	0.76	0.60–0.96	0.021
Laughing	0.80	0.64–0.99	0.042
**Random effects**			
σ^2^	3.29		
τ_00 Sequence_	2.59		
τ_00 Group_	1.06		
ICC	0.53		

CI, confidence interval; σ^2^, overall random effect estimated variability; τ_00_, random intercept variability for group and sequence; ICC, intraclass correlation coefficient.

Concerning laughing or leaning forward, the results suggest that there is a negative relationship to CC QCGE. The odds ratios indicate a 20% increase in the odds of being rated lower on CC QCGE for each one-unit increase in laughing. Concerning leaning forward, the odds ratios indicate a 24% increase in the odds of being rated lower on CC QCGE for each one-unit increase in leaning forward. Moreover, the random effects seem to explain parts of the variability of the model, supporting the inclusion of random intercepts of the group and the sequence.

The relationship between nodding and CC QCGE is exemplified in Table 11. In the activity transcript, there were high frequencies of nodding. For this group, the initial phase seems to have been successful in terms of high Social QCGE, and trust has been built, which can foster Cognitive and CC QCGE, as stressed by Sinha et al. (2015) and described above (section Indicators of Social QCGE). The group conversation shows a high-quality CC QCGE: group members link individual pieces of information together in such a way that an overall picture emerges. In this way, an attempt is made by the group to find an answer to the overarching question, namely, who the murderer was (see the sequence from min. 07:24.5). Group Members 1 and 2 gather and link pieces of information, and Group Member 3 helps them by asking questions and confirming their statements. These confirmation activities of Group Member 3 occur verbally (“mhm”) as well as non-verbally (head nodding).

**Table 11:** Section of action transcript of Group 2.

Time	Group Member 1	Group Member 2	Group Member 3
07:24.5		So, they have confirmed that he was at the tennis court at 7 a.m.	
07:30.1		[head nodding]	
07:31.2			Did they confirm that, or did he just say that?
07:32.4		No, they confirmed that, but it’s the cause of death, or the time of death could have been already at half past six, that means he could have killed him before he went to the tennis court…	
07:37.0			[head nodding]
07:40.4			[head nodding]
07:47.7	mhm… I read that Schmidt and Mr. Meier live only 10 min away from each other, by car…		
07:47.7			[head nodding]
07:51.6		[head nodding]	

## Discussion

In this study, the non-verbal behaviors of virtual synchronous student groups completing a complex problem-solving task in a CSCL setting were analyzed based on video recordings. Moreover, the quality of collaborative group engagement (QCGE) in these virtual groups was sequentially rated. Using a mixed-methods approach, we investigated two research questions: First, how do the student groups communicate non-verbally in the virtual synchronous learning setting? Second, which non-verbal behaviors are indicative of which dimension of QCGE?

Concerning the first research question, we found that a variety of non-verbal behaviors were displayed with different frequencies (see Table 1; Figure 1): In sum, the non-verbal behavior coded most frequently was group members` head nodding. The non-verbal behavior coded least frequently was gesturing. Other non-verbal behaviors include laughing, head propping, leaning forward, gesturing, putting one’s hand in front of the face, and changing seating positions. These were coded at similar frequencies across the behavior categories. Results thus indicate that nodding is a non-verbal behavior that occurs more often in the online-videoconference situation than other non-verbal behaviors, which reflects the synchronous CSCL setting at hand with group members sitting in front of their PCs and cameras and talking about the problem they want to solve while only their upper body parts are shown. Yet, results show, too, that despite the limitations of the camera setting not only head nodding but also other behaviors did occur. This indicates that the groups interacted non-verbally in complex ways besides talking. On this empirical basis with the variety of non-verbal behaviors at different frequencies we found, it is possible to search for non-verbal behaviors that could differentiate regarding QCGE.

Concerning the second research question, we searched for non-verbal behaviors that indicate QCGE, focusing on all the non-verbal behaviors that were found in our study, including nodding, laughing, head propping, leaning forward, gesturing, hands in front of the face, and changing seating positions. To model the relationship between non-verbal behaviors and QCGE, we first descriptively explored QCGE. We found that the groups show different variances in their levels of QCGE. Moreover, dimensions such as Behavioral and Social QCGE indicate a skew toward higher levels, whereas Cognitive and CC QCGE indicate a more uniform distribution. We then applied statistical modeling to explore the relationship between the non-verbal behaviors and QCGE. The cumulative-link mixed models suggest that certain non-verbal behaviors significantly relate to QCGE:

1. For Behavioral QCGE, results show that more frequent instances of head propping indicate a lower quality. This can be interpreted by the assumption that head propping may signal boredom and thus a greater tendency to be distracted or off-task. This is consistent with literature that associates head propping with boredom (Behoora and Tucker, 2015). However, in our virtual synchronous CSCL setting, head propping on the table in front of the webcam may also indicate that an individual is focused on the screen. The group members may be reading something, and in combination with leaning forward, head propping could be misinterpreted as off-task behavior. Nevertheless, the results of our analysis suggest that head propping is associated with lower-quality Behavioral QCGE. However, as the Behavioral QCGE ratings indicate a large ceiling effect, this finding should be taken with caution. Head propping may indicate moderate Behavioral QCGE ratings. However, it is not clear what non-verbal behavior may indicate higher levels of Behavioral QCGE other than lower frequencies of head propping.
2. Regarding Social QCGE, we found that no non-verbal behaviors relate significantly to this dimension. However, the odds ratio for head nodding at least suggests that more frequent instances of head nodding relate to higher quality. Moreover, the qualitative analysis suggests a certain face validity of this relationship as illustrated in the activity transcript (see Table 7). Therein, the group members ended each other’s sentences accompanied by frequent head nodding. Nevertheless, compared to a parallel study we ran investigating CSCL groups in a face-to-face setting (Paneth et al., 2024), the missing significance of the results in this study here is surprising. In the face-to-face setting of the other study, we found that head nodding relates significantly and positively to Social QCGE. Therefore, it is relevant to discuss what could be the reason for the lack of significance in our present study here. First, the low variance in Social QCGE and rather limited data could explain why the analysis does not suggest a relationship here. Second, the results may be due to reduced social cues in the virtual CSCL setting, similar to virtual synchronous communications described by Kiesler et al. (1984) in the theory of reduced social cues. This theory postulates that social cues like head nodding would be less easily perceived under virtual conditions than in face-to-face settings. In theory, one could argue that nodding relates to responsiveness, which is a criterion for high Social QCGE. As a consequence, in our study, group members might have felt less mutual reinforcement and responsiveness even though there was a verbal confirmation (e.g., “mhm,” “yes,” “ok”). According to recent literature (Hardwig and Boos, 2023), trust in groups decreases with increasing virtuality. In this respect, it may be particularly important in virtual synchronous learning group settings to build trust, which, in turn, can be supported by head nodding, especially regarding the reduced social presence that students experience in virtual synchronous settings (Ferri et al., 2020). Once “virtual trust” is established, group members can reach their full potential (Kazemitabar et al., 2022), and learning groups are more successful (Gerdes, 2010). This, in turn, is a good basis for the high quality of the other facets of engagement (i.e., cognitive and CC QCGE), which is in line with Sinha et al. (2015), who state that high-quality social QCGE at the outset of a group learning task can support other engagement facets as the task progresses. Even though there may be a lag between utterances, non-verbal behavior such as nodding still accompanies verbal communication, as used in a face-to-face setting. However, there is no evidence that the participants were looking at the videoconference or, therefore, at the non-verbal information the other participants would be signaling. Hence, the question is whether nodding is only a habit from face-to-face interaction and is still used even though the nodding may not be received by the other group members who do not observe the videoconference window. This aspect would be interesting to further investigate in future research.
3. Concerning Cognitive QCGE, results show that groups that exhibited more frequent laughing or leaning forward have significantly higher levels of QCGE. However, more frequent head nodding indicates lower Cognitive QCGE. Laughing as an indicator of Cognitive QCGE seems counter-intuitive. However, it seems that laughing can function as a facilitator to enhance joint regulation and monitoring. According to the action transcripts, during a task, when a group experiences a comical situation that triggers laughing, afterward the group jointly regulates the emotional outburst. This joint regulation may lead to a “restart” of the group processes and reorientation in the task progress. Therefore, an instance of frequent laughing may be indicative of subsequent higher-quality cognitive QCGE. In addition, as humor helps CSCL groups to lighten serious learning topics and thus make them more manageable (Hovelynck and Peeters, 2003), laughing can serve as a facilitator for the use of regulatory strategies such as planning and monitoring and thus for high-quality cognitive QCGE. Laughing could also be a form of social QCGE. As Sinha et al. (2015) also point out, good group cohesion or a high-quality social QCGE, which can be facilitated by laughing, is an important premise for high QCGE as it is also related to positive socio-emotional processes (Hu et al., 2021). However, laughing could also just be an indicator of off-task communication. Therefore, the subsequent regulation may just be the consequence of the disengagement from the task, which is identified by the laughing of the group members and not facilitated by it. In general, the direction of this laughing-to-joint relationship effect must be further explored and confirmed. In contrast to this result, the positive relationship between leaning forward and Cognitive QCGE is more intuitive. Leaning forward could be understood as a general form of engagement and interest in virtual synchronous settings, as is pointed out by related literature (Behoora and Tucker, 2015). Be it, to spend more focus on what is happening on the computer screen or, in a fashion, to be more rooted in a face-to-face setting, where the signalization of leaning forward could imply the direction of focus for a person in a group, or in this case the video conference window. Finally, head nodding relates negatively to Cognitive QCGE. This result is rather surprising, as one could argue that the nodding behavior could be indicative of co-constructive processes in relation to the regulation effort that is Cognitive QCGE. Thus, nodding would appear to be a function of socially shared regulation (Hadwin et al., 2017; Järvelä et al., 2018). Moreover, in a face-to-face setting, the relationship seems to be indeed positive (Paneth et al., 2024). Therefore, the nodding behavior seems to have a different function in a virtual synchronous environment. Furthermore, it could be argued that during instances of joint regulation and task monitoring, there is more need for verbal discussion than for non-verbal agreement through nodding. Moreover, participants may also engage in task-related behaviors such as overlooking current progress, monitoring the time left to accomplish the task, or making notes of the discussion. Especially in this virtual synchronous setting, a sequence of higher focus may lead to a higher focus on the task at hand and therefore away from the video conference, leading to less head nodding.
4. Finally, for CC QCGE, more frequent head nodding goes along with significantly higher ratings. The positive relationship between head nodding and CC QCGE may stem from the higher intensity of discussion during instances of high CC QCGE, which is consistent with related literature suggesting that head nodding fosters a smooth communication process (Allmendinger et al., 2003). The group is attempting to connect and share information to find the answer to the overarching question. In this case, the discussion leads to the formation of a thesis, which is confirmed by the other group members through verbal (e.g., “mhm,” “ok”) and non-verbal (i.e., nodding) statements. It seems that the head nodding has a reinforcing effect on CC QCGE in the sense that the group members feel confirmed and encouraged in their statements and thus have enough trust built to further elaborate their collaboration and deepen the conversation about the murder case. Finally, leaning forward and laughing negatively relate to CC QCGE. One could argue that high CC QCGE occurs more frequently closer to the finalization of the task, where information is shared and a conclusion will be made, which requires a lot of concentration. At the same time, there may be little time left for funny situations, and therefore, less laughing occurs. Over all four QCGE dimensions, the results suggest that different types of non-verbal behaviors have different indications of the four dimensions of QCGE. Most prominently, the non-verbal indicators for Cognitive and CC QCGE are the same, but with opposite directions of the relationship. Specifically, laughing is an indicator of higher cognitive QCGE and lower CC QCGE. Furthermore, leaning forward and head nodding indicate higher CC QCGE and lower Cognitive QCGE. Thus, the combination of these three non-verbal behaviors may distinguish between CC and Cognitive QCGE. Moreover, head nodding can indicate higher Social QCGE but may also indicate lower Cognitive QCGE. From there, different combinations of non-verbal indicators could be used to more reliably indicate the different dimensions of QCGE.

### Limitations

This study has its limitations. The sample size would be low for the investigation of group-level differences. However, the use of repeated measurements for both QCGE and non-verbal behavior facilitates that limitation. Nonetheless, the results of this exploratory study should be confirmed, optimally with a larger sample size. Moreover, our correlational analysis restricts us from interpreting the causality of the relationships we have found between non-verbal behavior and QCGE dimensions. Future research could apply a more experimental approach to investigate causality. Based on this research, directed hypotheses could be formulated and tested to confirm the relationships we found exploratively.

Regarding Behavioral QCGE, the rating of the on-task behavior was additionally challenged because the raters were not able to fully deduce from the transcripts whether the participants were on- or off-task. Presumably, some participants were just reading something on the screen while still being on-task. This limits the validity of the Behavioral QCGE rating and may also explain the low variance and skew toward higher ratings. Moreover, for Social QCGE, the study setting may incentivize pro-social behavior and social desirability effects and therefore fewer instances of low or moderate Social QCGE scores, which could also impede the study of this variable. Concerning the QCGE framework, in other research we report issues with the QCGE rating scale, which can lead to skewed Behavioral and Social QCGE (Paneth et al., 2023). We assume that the rating scale may not be optimal for standardized study settings where groups are incentivized to consistently be engaged during the task. This resulted in a lower variance for Behavioral and Social QCGE, making it difficult to model this variable.

### Implications

In general, the results of this study show how student groups communicate non-verbally in a virtual CSCL setting through a set of complex non-verbal behaviors, and they show that certain non-verbal behaviors are related to different dimensions of QCGE. Similar findings were reported in a prior study by our research group, conducted in a face-to-face setting (Paneth et al., 2023, 2024). What are the implications of the results?

Regarding theory building, the results on the non-verbal behavior of CSCL groups in videoconferencing add to our understanding of QCGE as a complex construct in need of further theory development (see also Rogat et al., 2022). The results from our study show that QCGE “in the field” and even in a virtual setting is not only located in the thinking and verbal communication of learners but manifests itself in their bodily communication (non-verbal behaviors, e.g., differentiating between Cognitive and CC), and this extends and substantiates the construct of QCGE in line with both theories of social interaction (e.g., Burgoon and Dunbar, 2018) and embodied cognition (Varela et al., 2017; Gallagher, 2018). This, in turn, is important for further theory building, future research on QCGE, and, generally, investigations aiming at better understanding and assessing group processes.

Regarding methodology, we present potential new measures for QCGE (i.e., non-verbal indicators) in line with our prior multimethod approach (Paneth et al., 2023). Moreover, based on our findings, we can suggest that certain non-verbal indicators for QCGE could be potentially automatically measured to assess collaborative engagement more accurately. Head nodding has already been established as a non-verbal behavior that can automatically be classified (Nguyen et al., 2012). The authors successfully applied a multimodal approach based on a combination of visual and auditory signals. In addition, leaning forward and laughing would presumably also be feasible to automatically detect in video recordings. With modern open-source frameworks like MediaPipe (Lugaresi et al., 2019), models can be trained to identify smiling faces and body poses from video recordings. With the combination of laughing, leaning forward, and head nodding, an automatic differentiation between Cognitive and CC is promising. Regarding Social and Behavioral QCGE, more research should be conducted to generate more data and variance to explore the intricacies of these two dimensions. Then, non-verbal behavior could be identified that would differentiate between the QCGE dimensions and the levels within. Combined with automatic transcription of verbal communication, verbal indicators of QCGE (Jeitziner et al., 2023, In preparation)^1^ (Zheng et al., 2023) could add more information to build a holistic measure of QCGE.

This leads to practical implications of our findings for educational use. This granular multimethod measurement and possibly automated measurement processes would increase the chances of applying targeted and effective interventions for virtual synchronous CSCL groups. While we are careful not to overgeneralize and overinterpret our findings (see section Limitations), we do suggest supporting the design of virtual learning environments in line with related research. As suggested by CSCL researchers (e.g., Järvelä and Hadwin, 2013), students need regulation in CSCL, either through teacher or lecturer feedback to groups to support socially shared regulation of learning or “from the outside” through CSCL tools, e.g., in the form of scripting or scaffolding (Vogel et al., 2017). A detailed knowledge of QCGE and how it is expressed in non-verbal behaviors could support this, e.g., through real-time analysis and feedback systems (Deeva et al., 2021; Zheng et al., 2023) that automatically analyze and mirror groups’ QCGE and allow students and teachers to become more aware of group processes and thus regulate them (Hmelo-Silver and Jeong, 2023). Our results could lead to important original insights for the design of such feedback systems. Some initial, more far-reaching implications include that practitioners who use virtual group learning settings are aware of the QCGE dimensions (Behavioral, Social, Cognitive, and CC) and the importance of non-verbal indicators. If practitioners knew more about the complexities of collaborative group engagement, for example, through teacher education or training, they could differentiate what student collaborative engagement is and how it manifests and develops their own interventions.

In conclusion, this study contributes to the development of the assessment of QCGE in virtual synchronous CSCL groups and could serve as a basis for real-time feedback to promote QCGE during virtual synchronous courses at universities. The analysis suggests that the non-verbal behavior of CSCL groups in videoconferencing may be a promising aspect for further investigation to understand and assess group processes.

## Data availability statement

The anonymized data and analysis scripts supporting the findings of this study are available at the Open Science Framework (OSF) repository via the following link: https://osf.io/kngdx/.

## Ethics statement

The studies involving humans were approved by Ethics committee, School of Applied Psychology, University of Applied Sciences and Arts Northwestern Switzerland. The studies were conducted in accordance with the local legislation and institutional requirements. The participants provided their written informed consent to participate in this study.

## Author contributions

LJ: Data curation, Formal analysis, Methodology, Visualization, Writing – original draft, Writing – review & editing, Investigation, Software. LP: Conceptualization, Methodology, Writing – original draft, Writing – review & editing, Investigation, Project administration, Resources, Visualization. OR: Conceptualization, Funding acquisition, Supervision, Writing – original draft, Writing – review & editing, Resources. CZ: Conceptualization, Funding acquisition, Project administration, Supervision, Writing – original draft, Writing – review & editing, Resources.

## Footnotes

^1^Jeitziner, L., Paneth, L., Rack, O., Zahn, C., and Wulff, D. U. (2023). Exploring Linguistic Indicators of Social Collaborative Group Engagement. In: Proceedings of the 16th International Conference on Computer-Supported Collaborative Learning—CSCL 2023. International Society of the Learning Sciences. Eds. C. Damsa, M. Borge, E. Koh and M. Worsley. 364–365. doi: 10.22318/cscl2023.813595.
